# Towards smart biomanufacturing: a perspective on recent developments in industrial measurement and monitoring technologies for bio-based production processes

**DOI:** 10.1007/s10295-020-02308-1

**Published:** 2020-09-07

**Authors:** Carina L. Gargalo, Isuru Udugama, Katrin Pontius, Pau C. Lopez, Rasmus F. Nielsen, Aliyeh Hasanzadeh, Seyed Soheil Mansouri, Christoph Bayer, Helena Junicke, Krist V. Gernaey

**Affiliations:** 1 Process and Systems Engineering Center (PROSYS), Department of Chemical and Biochemical Engineering, Technical University of Denmark, Kgs. Lyngby, Denmark; 2 Department of Process Engineering, TH Nuremberg, Nuremberg , Germany

**Keywords:** Bioprocesses, Industry 4.0, Big data, Control, Sensors, Smart biomanufacturing

## Abstract

The biomanufacturing industry has now the opportunity to upgrade its production processes to be in harmony with the latest industrial revolution. Technology creates capabilities that enable smart manufacturing while still complying with unfolding regulations. However, many biomanufacturing companies, especially in the biopharma sector, still have a long way to go to fully benefit from smart manufacturing as they first need to transition their current operations to an information-driven future. One of the most significant obstacles towards the implementation of smart biomanufacturing is the collection of large sets of relevant data. Therefore, in this work, we both summarize the advances that have been made to date with regards to the monitoring and control of bioprocesses, and highlight some of the key technologies that have the potential to contribute to gathering big data. Empowering the current biomanufacturing industry to transition to Industry 4.0 operations allows for improved productivity through information-driven automation, not only by developing infrastructure, but also by introducing more advanced monitoring and control strategies.

## Introduction

Industry 4.0 and the smart manufacturing movement now provide biomanufacturing the opportunity to upgrade its production processes to be in harmony with the latest industrial revolution [118]. Due to the promise of increased productivity and flexibility, there is significant interest from both managers and process engineers to transform their plants to smart manufacturing facilities. However, in many companies, manufacturing is usually one of the last parts to embrace innovation, since doing so might come with great investment, expensive downtime, and prolonged licensing and regulatory updates. Facing this risk, management has to be convinced of the real and distinct benefits that can be achieved by implementing real innovation on the production floor.

One of the trademarks of Industry 4.0 is *big data*, which refers to large sets of process and product data collected by sensors and process analytical technologies (PAT) [1]. Among several other benefits, integration of data from operations and business activities can promote productivity by allowing greater visibility across upstream and downstream operations. Being able to use historical and real-time data to predict future outcomes is an empowering tool that can help employees to be proactive instead of reactive. They can understand in an agile manner what is happening in a process and why, as well as predict what will happen when variations occur [2]. The gains obtained from such a proactive, predictive feed-forward control approach can exceed the incremental yield improvements that companies seek.

Besides bringing transparency and more informed decisions, being able to use these data will also allow the factory to, for example, easily adapt to schedule and product changes in a way that requires minimal intervention, allowing swift changeovers and decreased cleaning validation times.

Furthermore, these data can be used for optimization purposes by applying advanced big data analytics. Machine Learning (ML), a branch of Artificial Intelligence (AI), is one of the ways to achieve this. ML works with small to large datasets by analyzing and comparing the data so as to find mutual patterns and explore differences [3].

Being able to rely on automated systems that require minimal human/manual intervention will result in higher yields and quality, alongside with decreased costs and waste generation, which is of great importance to bio-based production, and especially biopharma.

Although hesitant to implement AI/ML techniques due to strict requirements for GMP compliance [4], the biomanufacturing industry has a lot to benefit from data analytics. Data analytics are the key to provide real-time insights, as well as enabling evaluation and validation of all critical process parameters against regulatory guidelines, ranging from raw materials to the finished product. This actually helps companies, especially within the biopharma sector, to comply with the strict and compulsory requirements that are characteristic of that sector.

Nevertheless, despite this positive outlook, there are technical, economic and organizational challenges, and likely some unknowns, that must be addressed to successfully implement these technologies in biomanufacturing.

However, the most significant obstacle towards the implementation of smart manufacturing in bio-based industries is the collection of large sets of relevant data. The current situation is that many companies are still in the process of shifting from manual to automated systems (Industry 3.0) [4].

Hence, there is still a long way to go for many biomanufacturing companies to fully benefit from smart manufacturing as they need to transition their current operations to a data-rich future first. Thus, the objective of this manuscript is both to summarize recent advances with regards to the monitoring and control of bioprocesses, and to highlight some of the key technologies that have the potential to overcome the aforementioned limitations, which have, so far, prevented smart manufacturing from being fully realized in the bio-based industry. By developing the required infrastructure as well as data-driven monitoring and control-strategies, this transition of the current biomanufacturing industry to Industry 4.0 operations allows for improved productivity while ensuring regulatory compliance.

## State of the art

Smart factories hold the promise to also increase sustainability through real-time monitoring of production, where the automated control systems are expected to reduce the number of faulty batches and cut the maintenance costs. Thus, the aptitude of biomanufacturing companies to automatically and appropriately control the bioprocesses in their optimal state is of crucial importance, as this helps to reduce or maintain the production costs and increase yields, while keeping the uniformity of product quality. The technology readiness level (TRL) of advanced sampling methods and automated measurement techniques may significantly reduce the time needed for process monitoring and control. This is emphasized by the FDA, which highlights the use of new monitoring and control approaches, such as Process Analytical Technology (PAT) tools, to improve and guarantee product quality, particularly in the pharmaceutical industry [5]. Noteworthy is that, a great part of the PAT objectives are general in nature and thus can be applied not only to pharma but also to any biomanufacturing process [6].

Carrying out the effective implementation of PAT, or any other monitoring and control approach, depends on, among other things, the availability of robust and reliable sensors, and full understanding of the intrinsic variability of bioprocesses [7]. Thus, this framework requires process understanding based upon scientific knowledge aiming at monitoring and control of all critical process parameters that affect the quality of the final product [8]. To this end, PAT consists of tools that include design of experiments, bioprocess modelling, multivariate data analysis and sensor technologies. Studies presented along the years, such as [9, 10], have shown that there are great benefits behind developing mathematical models especially for the optimization and control of bioprocesses. The development and implementation of modelling strategies, real-time monitoring, optimization and control, is required to ensure operational reproducibility, quality control and consistency [11]. However, although being aware of the potential benefits, in the biomanufacturing industry the processes are still vastly optimized and controlled without the explicit use of these models*.*

### Monitoring and control of bioprocesses

**Fig. 1:**
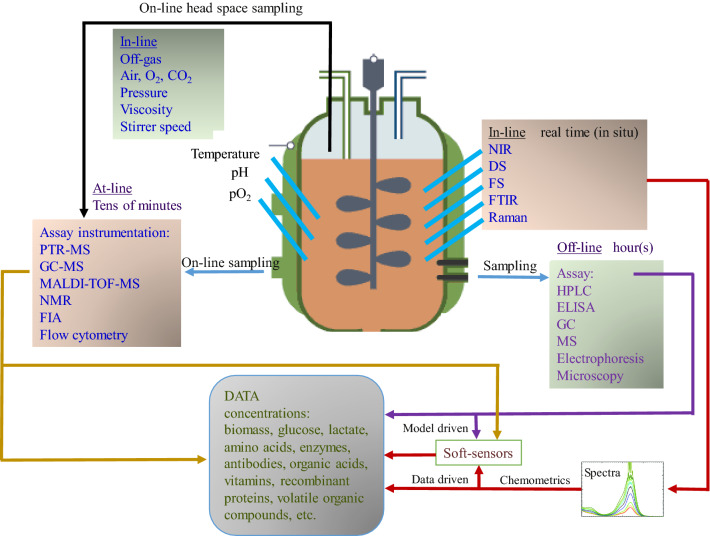
Schematic of bioprocess monitoring: variables and different analytical techniques. *NIR* near-infrared spectroscopy, *DS* dielectric spectroscopy, *FTIR* Fourier-transform infrared spectroscopy, *FS* fluorescence spectroscopy, *HPLC* high-performance liquid chromatography, *ELISA* enzyme-linked immunosorbent assay, *GC* gas chromatography, *MS* mass spectrometry, *PTR-MS* proton transfer reaction mass spectrometry, *MALDI-TOF-MS* matrix-assisted laser desorption ionization time-of-flight mass spectrometry, *NMR* nuclear magnetic resonance, *FIA* flow-injection analysis. Not all methods can be deployed in an on-line fashion. Adapted from [11]

Notwithstanding the different objectives and end products, most biomanufacturing processes include the cultivation of microorganisms, which implies a process consisting of complex chemical, physical and biological phenomena [12]. A dependable and consistent analytical system is necessary to control the process conditions in all parts of the biomanufacturing process (upstream, downstream and product formulation). For example, the upstream processing part, once it includes cell growth, is a complicated multi-phase system with a considerable variability due to the inherent nature of the cell cultures. Hence, sensors are required to measure physical variables such as temperature and pressure, chemical quantities such as pH and dissolved oxygen as well as biological parameters such as cell density or metabolite concentrations (Fig. 1). It may be noted that most biological variables are particularly difficult to measure and monitor.

**Fig. 2:**
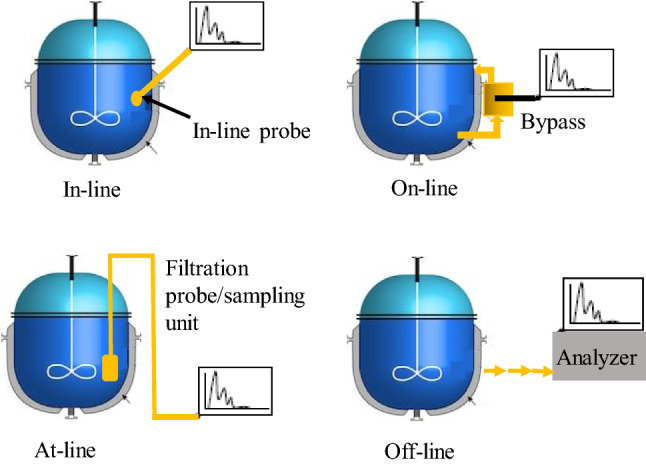
In-line, at-line and off-line sensors. Adapted from [8]

Monitoring methods and their associated sensors and analyzers, can be further categorized according to their position regarding the process unit as in-line, at-line or off-line as illustrated in Fig. 2.

An in-line sensor produces data continuously (no sampling), and it is in direct contact with the process medium (invasive) or separated from the medium by a glass window (bypass, non-invasive, also called on-line sensor). By providing continuous information, these sensors are enablers of continuous process control.

At-line sensors analyze samples in close proximity to the bioreactor. Even though the samples are collected at regular time-intervals (manually or automatically), time delays due to the analysis (depending on the equipment) render such data suitable for monitoring purposes, but not for control. Finally, samples for off-line measurements are collected manually or automatically and then transferred to the laboratory to be analyzed. This causes long time delays, so that such measurements cannot contribute to the control of the dynamic process behavior [8, 12].

#### Towards real-time data collection

Real-time monitoring of bioreactors is seen as a crucial part of effective bioprocess control since it can help achieve high efficiency, productivity and reproducibility [12, 13]. Although seldom available, timely process information about biomass, substrates, metabolites, products and nutrients is vital to take effective control decisions [11, 14, 15]. Furthermore, measuring products such as cells, proteins and by-products is crucial to guarantee that the production process is developing as expected. The opportunity to assess how the fermentation is progressing makes it possible to not only optimize the process by promptly regulating specific parameters, but also to spot when the process is not under optimal operation [11, 16].

In the particular case of fermentation processes, additional measurements may be used to measure variables such as dissolved CO_2_ (in the off-gas) and optical density. For example, in the case of cell density, it is usually measured using optical in-situ probes, or chemometrics coupled with intact cell mass spectrometry (ICMS) [16], impedance spectroscopy [17], near infrared spectroscopy (NIR) or Fourier-transform-infrared spectroscopy (FTIR) [18].

Furthermore, combined analytical and chemometric approaches are being developed to monitor the substrate, metabolites and products in bioprocesses. Chemometrics are employed to provide real-time analysis of variables that otherwise would require off-line analysis and interpretation using methods such as HPLC or GC/MS [19]. A few examples include in-situ biosensors [20–22], optical sensors [23, 24], at-line implementations of traditional methods (HPLC, GC/MS) [19, 25] and spectroscopic sensors [26–42], among others.

These combined analytical and data-driven approaches are promising new developments that have the potential to monitor key process parameters in real-time. Therefore, they will be discussed in more detail in the following sub-sections.

#### In-situ biosensors

Biosensors are analytical tools consisting of an immobilized sensing material in close contact with a suitable transducer that converts the biochemical signals into quantifiable electrical signals [43]. A typical biosensor includes three parts, an immobilized biological detection element on a signal transducer unit, which is amplified by a signal conversion unit.

The analytes are sensitively and selectively recognized by the bio-components either via a catalytic mechanism (e.g. enzymes, cells, tissues, organelles, etc.) or through binding (e.g. protein channels, antibodies, nucleic acids, etc.). The interaction of the biological detection element and the analyte is determined by the transducer unit. This unit can use optical [44], electrochemical [45], calorimetrical [46], or piezoelectrical principles [47]. The integration of biological elements such as enzymes, microorganisms, and antibodies as sensing materials makes the transducer selective and sensitive. Due to their properties, these sensors have the capability of providing fast, cost effective and reliable analytical results [43], which together with expanding computation power, enables the use of biosensors as another promising solution enabling the drive towards smart manufacturing. Recently, several biosensors have been developed for the control and monitoring of many different analytes [44, 48, 49]. In particular, biosensors for the on-line determination of glucose, glutamate, and lactate are available [49, 50], which are delivered ready to use and can be directly integrated with bioreactors via standard ports. However, these sensors are still in the development stage and are not used in industrial scale production processes.

#### Spectroscopic sensors

Spectroscopic sensors are quite promising for successful bioprocess monitoring for two reasons. Firstly, nearly all biological, chemical and physical variables are accessible by spectroscopy using the whole spectrum (from UV to MIR) [51]. Secondly, as non-destructive in-line sensors they provide information with little or no time delay, thus enabling real-time monitoring and control of several process variables [12, 51, 52]. A good example of the implementation of spectroscopic sensors in biomanufacturing is the in-line use of 2-D fluorescence spectroscopy which was applied in a high-throughput fermentation system called BioLector^®^. This system enables the monitoring of different microorganisms and mammalian cells, fluorophores (e.g., GFP, YFP), and NAD(P)H [53, 54].

Even though spectroscopic sensors fall in line with the PAT initiative [55] and seem to be a promising step towards smart manufacturing, there are far more applications of spectroscopy published in research than in industry. There are several reasons behind this: (i) high requirements for well-documented analytical systems especially in GMP manufacturing environments; (ii) many industries do not publish the details of the monitoring procedures used; and, (iii) great investment involved in its industrial implementation, both in terms of equipment and in terms of the requirement of highly qualified personnel to set up and maintain such an instrument. Some companies have attempted to tackle the need for qualified personnel in the industries by developing solutions that give a third party access to the data, such as AnalyticTrust from Q-Interline [56], where the quality of the analytical instrument and the data it generates is monitored by a third party. However, the security issues are a usual argued obstacle as to fully embrace these new technologies, and it is important to ensure that all data are protected sufficiently.

#### Chemometrics (multivariate data analysis)

Spectroscopic sensing produces large datasets and thus requires chemometrics, or multivariate data analysis (MVDA), to be able to provide continuous real-time monitoring of the bioprocess variables. MVDA is a subset of machine learning algorithms that deals with multiple variables simultaneously. MVDA is used to extract information from the spectra, by processing the data and reducing the complexity of a data set. To this end, data pre-processing is a powerful tool that can reveal relevant information in the data [8, 12, 57]. After pre-processing, model calibration is performed so as to retrieve qualitative and quantitative information from the spectral data. Many MVDA approaches are based on Principal Component Analysis (PCA), which is often used to investigate the structure, variance and/or distribution of the dataset and to identify outliers [58]. As a qualitative approach and consistent with the PAT initiative [59]**,** PCA has been used for process supervision, to classify raw materials and batches, as well as to deduce the process status based on the spectral data [58, 60]. In this way, a process target line or trajectory, also called golden batch, can be identified from similar and ideal process runs [61–63]. Quantitative models, most often using partial least square regression (PLS) or sometimes neural networks, are used to characterize correlations between process variables and spectral data [8], and thus different variables can be predicted on-line from these measurements. This provides a thorough view of the process and enables process automation and rapid fault detection by closed loop control [8].

For example, in the case of fermentation, in regression models such as PLS, accounting for the linear relationship between the spectral data and the concentrations is very important due to the fact that many highly correlated compounds contribute to the spectral matrix. Thus, by constraining the I/O relation to a linear system, it is possible to make PLS models predict on causal relation, i.e., the absorption peaks of each pure compound are reflected in the latent structures of the PLS. This is important when the model is expected to be used in systems were these correlations can change.

After the model calibration, external validation is necessary so as to ensure that the developed model is as accurate as possible and behaving as expected. The model developed should adapt to the process and measurement conditions, thus changes in the actual process such as raw materials or equipment require model updates [8]. Model changes and updates require highly qualified personnel, and therefore, third parties such as Analytic Trust [56] are often seen as quite relevant and necessary to provide these resources.

As highlighted, new opportunities for automation, monitoring and control are closely related to the use of machine learning algorithms to interpret the large quantities of data collected and return intelligent information.

#### Free‐floating wireless sensors

Recently, floating sensor devices without a physical connection to a reactor have been proposed. These floating sensors follow the flow in the bioreactor and collect data along a trajectory, transmitting it by wireless technology [64]. Zimmermann et al. [65] developed the wireless spherical particles for the determination of fluid dynamics. This type of sensors are already commercially available (e.g., smartINST, smartCAPS, France, and Freesense, Denmark) in the form of spheres. These spheres are deployed inside the bioreactor and move around freely to measure variables such as pH, temperature, pressure, conductivity, and turbidity during production. The sensor spheres are composed of a sterilizable shell, electronic boards, a battery, and the sensing element. They gather data on several variables (e.g. temperature, pH, pressure, dissolved oxygen) simultaneously and then transmit this information in real-time to an external data analysis unit. These mobile wireless sensing devices offer significant potential for real-time monitoring and control of bioprocesses, not only due to the on-line data collection and analysis, but also due to the fact that these data represent real gradients inside the reactor. While these sensors would not be used during all production campaigns, they can be used during selected ones to monitor the spatial evolution of a reactor (fermenter) and hence provide valuable insights so as to carry out operational changes.

#### Soft sensors

Soft sensors are advanced process monitoring systems, which use algorithms to assess measurements in an on-line manner to generate information about an otherwise unmeasured process state [6, 66]. Recently, they have been the focus of many studies since they appear to be an alternative to the traditional automated approaches, enabling the monitoring of state variables that indeed affect the bioprocesses but cannot be measured in real-time [67–70]. Spectroscopic sensors have, in some occasions, been labeled ‘soft sensors’ in the bioprocessing literature due to the fact that spectroscopic data is modeled using software and these models produce information similar to hardware sensors [57, 66, 71].

Three modelling approaches can be used for the design of soft sensors: (i) mechanistic models based upon first principles (white box) [10, 72–74]; (ii) data-driven models (black box) [58, 61, 75, 76]; and, (iii) hybrid models (grey box) [77–81].

The growing computer capacity and the advances in signal processing (AI and ML algorithms) have made soft sensors very convenient and enticing to monitor and control industrial manufacturing. Hence, the opportunities behind their implementation perfectly align with the PAT initiative and the smart manufacturing movement. Examples and more details on the development and implementation of soft sensors in the biomanufacturing industry are given in [6, 66].

## Enabling smart biomanufacturing

As illustrated in the state of the art section, there are recent developments in sensors, process monitoring and closed loop process control that are enabling the drive towards smart manufacturing in the biotech industry. The objective of this section is to present three specific examples of smart solutions developed by the team of authors at the Technical University of Denmark in close collaboration with industrial partners.

### Case study 1: monitoring of fermentation processes by imaging and image analysis

The assessment of microbial growth and the quantification of biomass, respectively, is the most evident way to evaluate the progress of any fermentation. The microbial biomass as such is the producing core of any fermentation process and frequently, the biomass is the product itself. However, biomass assessment and quantification are mostly limited to off-line analysis. Even if on-line growth detection systems may be applied, their performance is impaired during the very early and late stages of the fermentation, when very low and very high biomass concentrations are present, respectively.

The current status-quo method for growth detection is the measurement of the optical density (OD). The increase in absorbance, typically measured at 600 nm, represents the progress of the biomass formation. However, such measurements are compromised by a narrow linear OD detection range of 0.1–1, which equates to a cell dry weight concentration span of approximately 0.1–1 g/l. Besides, the limited reproducibility between different spectrometers also affects the results obtained by such measurements. To this end, there is an apparent need to identify alternative methodologies that provide better detection of these critical state parameters to make smart manufacturing a reality.

Recent progress in microscopic imaging and image analysis have opened an optical window into the reactor, so that cellular events may be observed and microbial growth be evaluated automatically. Microscopy has grown into an efficient tool establishing a basis for novel image-based monitoring and future control strategies. The imaging of living cells has yielded tremendous insights into cellular growth, functions and responses to environmental changes, for instance through information about cell size, shape, position and motility [82–84]. Challenges arising from different microscopic techniques such as bright-field [85] and fluorescence microscopy [86] that result in poor counting statistics, for instance a small field of view or the visualization of objects that are transparent on the image, so called ‘phase objects’, are solved by phase contrast [87] and confocal [88] microscopy. Yet, they are expensive, require dedicated infrastructure, have long acquisition times and, in addition, improper settings can cause significant artefacts on the images [89].

The oCelloScope instrument (BioSense Solutions ApS, Farum, Denmark) is a novel and compact solution based on bright-field, digital [90] and time-lapse [91] microscopy. The instrument facilitates a magnification factor of 200 and hence, enables the detection and segmentation of objects with a size between 0.5 µm and 1 mm. Accordingly, it is suitable for the investigation of mammalian cells, yeast/fungi, bacteria and crystals in (semi) transparent substances. The software for operation and analysis includes several state-of-the-art algorithms facilitating automated image acquisition and robust analysis. Objects are identified from segmented images by their true shape, while key properties such as surface area, perimeter and circularity are assigned.

The open software platform in use has been shown to provide considerable advantages in several research fields including microbiology [92], medicinal chemistry [93], pharmaceutical biotechnology [94] and basic cancer research [95]. Moreover, the technology has found a solid application area in both monitoring of bacterial growth and growth inhibition [96], respectively, as well as for detection of changes in microbial morphology [97].

The ability to measure microbial growth and to detect morphological features simultaneously render this system particularly attractive. For example, information about the cell size and cell size distribution in yeast cultivations have been shown to be correlated with the cell viability (dead/alive, osmotically stressed [98, 99]) and the growth rate of the culture [100]. Furthermore, the cell size was recently correlated to the accumulation of an internal product (fatty acids) in microalgae [101]. Consequently, image analysis seems to be a powerful tool that provides snapshots of the physiological state through the assessment of morphological features. Ultimately, image analysis can be used to assess the most crucial parameters such as microbial growth, substrate and product levels at a specific point in time, which forms the basis for novel image-based control strategies for fermentation process operations.

**Fig. 3:**
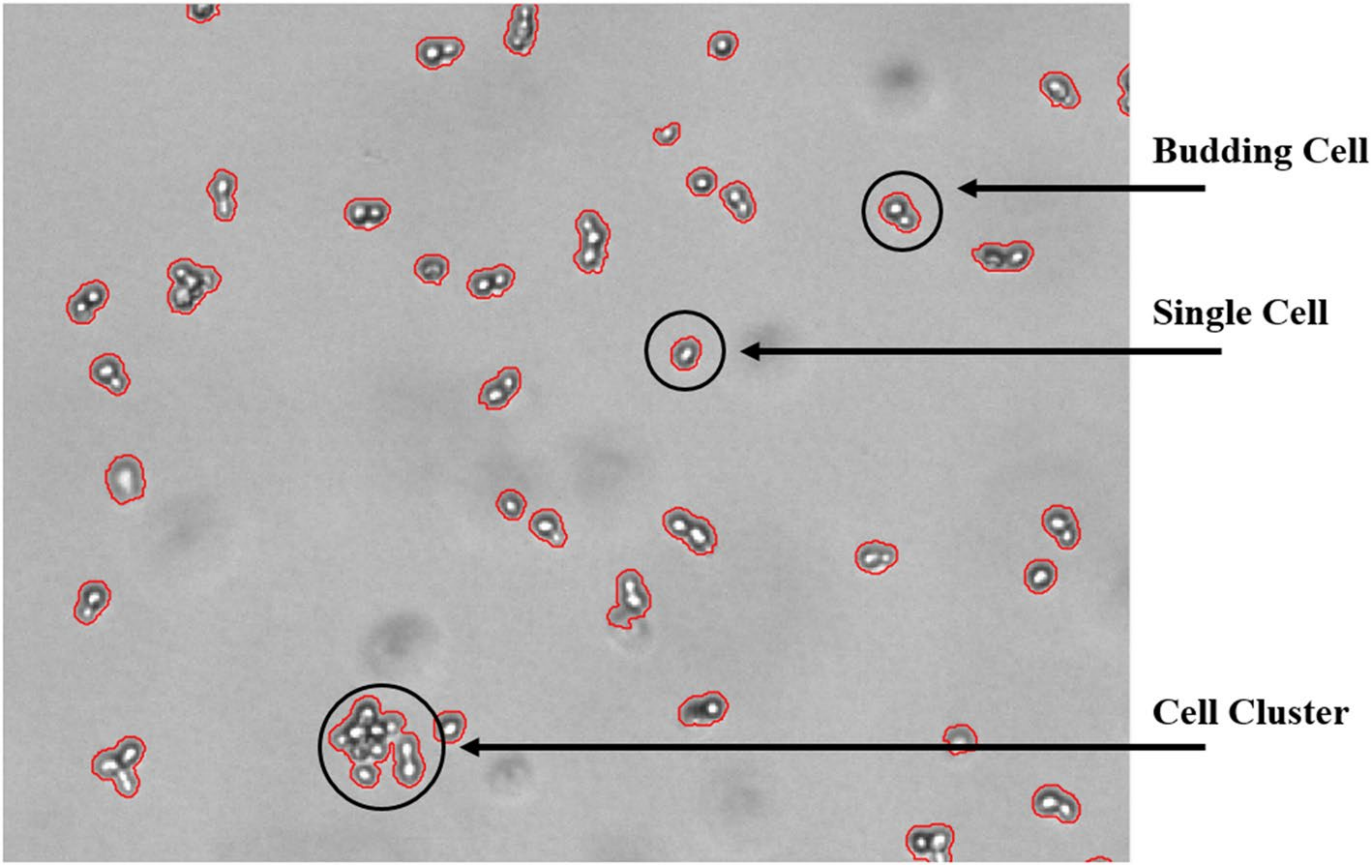
Image of a yeast cell culture possessing an OD value of approximately 0.1, grown in YPD medium. Yeast cells appear as a bright spot surrounded by a darker border. The bright spot feature counts the number of bright spots per object. As indicated in the figure, one bright spot represents a single yeast cell, two bright spots represent a budding yeast cell and three and more than three bright spots represent a cluster of yeast cells

Thus far, the application has been limited to microscopic slides and microtiter plates allowing off-line sample analysis only. However, the recent development of a flow-through cell as an alternative sampling device by ParticleTech ApS enables real-time, automated on-line image data acquisition and analysis. Hence, by means of the developed prototype flow-through cell, a *very first trial* of an on-line, image-based monitoring approach of a yeast fermentation process was set up by connecting the oCelloScope via a recirculation loop to the fermenter [102]. This first trial aimed at both growth detection and the evaluation of morphological trends. For automated distinction of single cells, budding cells and cell clusters, the so-called bright spot feature was developed and integrated into the software, exploiting the fact that the yeast cells appear as a bright spot surrounded by a dark border on the images. By counting the number of bright spots associated to a cell object, the bright-spot algorithm allows the automatic distinction between single cells, budding cells and cell clusters. An image of a yeast culture with different cell objects, acquired with the oCelloScope instrument, is depicted in Fig. 3.

**Fig. 4:**
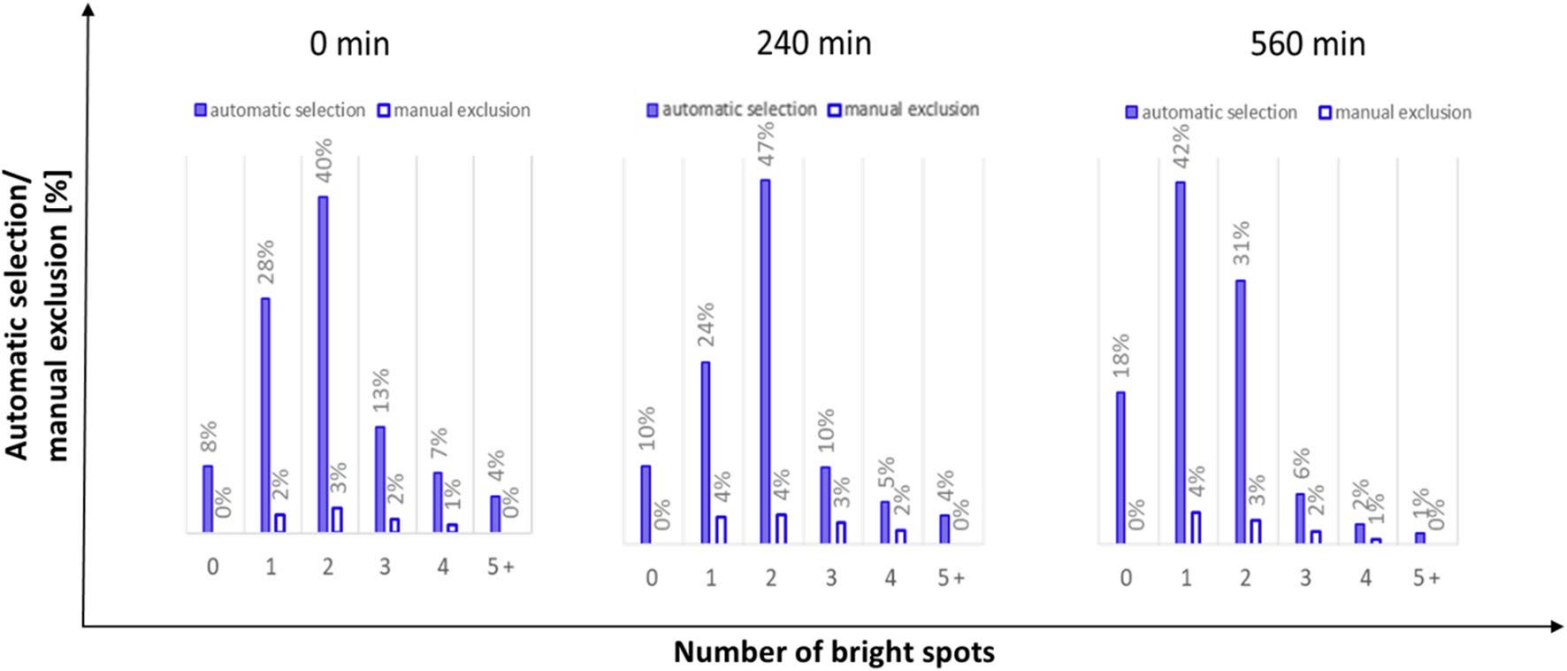
Evaluation of the bright spot feature exemplarily shown on images acquired after 0, 240 and 560 min of a lab-scale yeast fermentation process. The figure at time point 0 (inoculation) is to be interpreted as follows: 8% of all objects segmented on the image were automatically classified to have zero bright spots, out of which 0% were manually selected and excluded due to false segmentation. 28% of all objects segmented on the image were automatically classified to have one bright spot, out of which 2% were manually selected and excluded due to false segmentation. The other bars in the bar charts have to be read accordingly

The bright spot feature was demonstrated to work reliably with a failure rate of less than 5%, on average, as shown in Fig. 4.

The validation results shown in Fig. 4 are based on a lab-scale yeast fed-batch fermentation experiment (2 l working volume, YPD medium, grown at 30 °C, 800 rpm, 1 vvm, controlled at pH 6), with glucose addition (100 ml of a 400 g/l glucose solution) after 6 h. In parallel to the images collected with the oCelloScope instrument, samples from the reactor were withdrawn manually and diluted to an OD of approximately 0.1. The yeast cell concentration correlated to this OD value was previously investigated to yield an appropriate image quality, with respect to the separation of cell objects. On average 1300 cell objects were segmented on each image, and the results of three images [after inoculation (0 min of fermentation time), 240 min and 560 min of fermentation time] are displayed in Fig. 4. After automated classification into groups of cell objects exhibiting 0, 1, 2, 3, 4, and 5 (or more) bright spots, the groups were manually screened for false objects and any false object was manually excluded from the respective group.

Furthermore, the bright spot feature is capable of excluding image artefacts that result from shadings or out-of-focus cells in the background, which result in objects with zero bright spots.

**Fig. 5:**
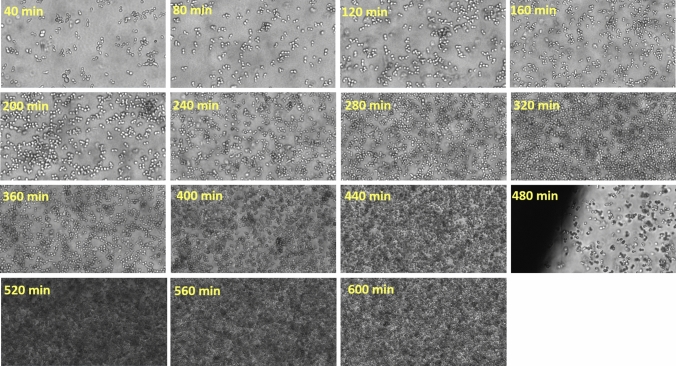
60% zoom into the images acquired on-line, and the relative time point of image acquisition is indicated for each image. The big shading on the left corner on the image acquired at 480 min resulted from the border of an air bubble which was temporarily stuck inside the flow-through cell

The application of the prototype flow-through cell effectively demonstrated the use of this technology for automated on-line growth detection. Images were collected every 10 min and a selection of these images acquired with the help of the prototype flow-through cell are shown in Fig. 5.

**Fig. 6:**
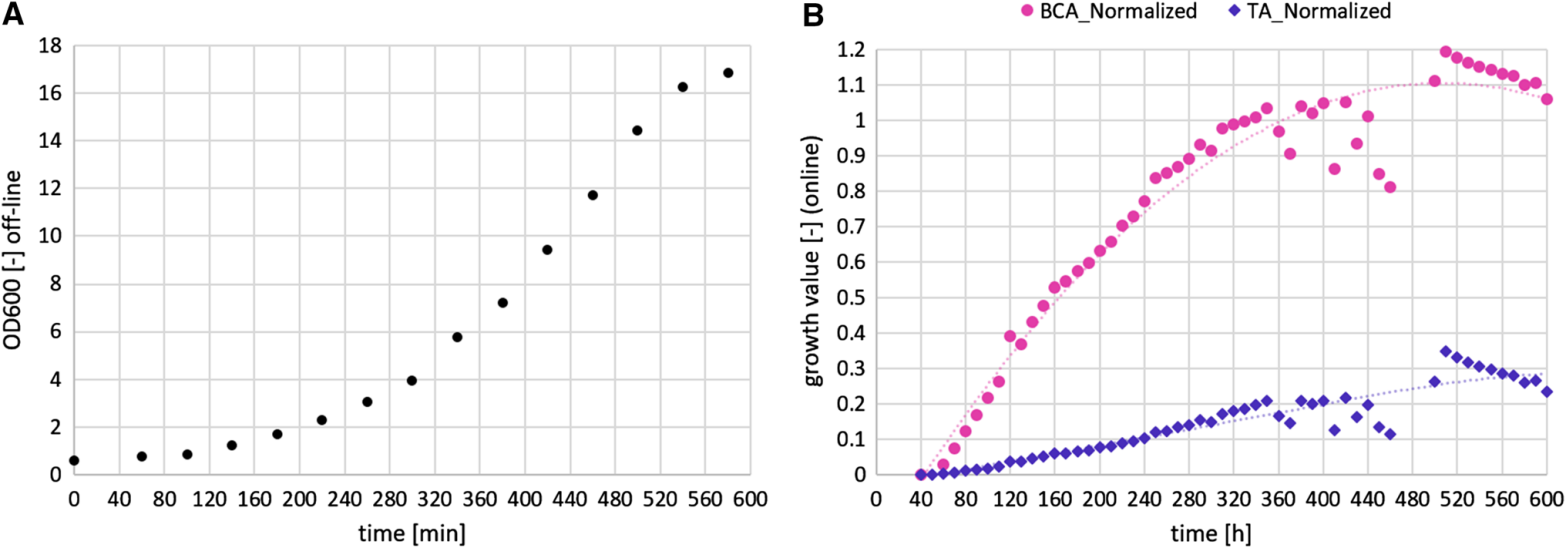
The increase of biomass followed by **a** off-line OD600 measurements, and **b** on-line via the normalized BCA and TA algorithm during a lab-scale yeast fermentation process (2 l working volume, YPD medium, grown at 30 °C, 800 rpm, 1 vvm, controlled at pH 6)

However, the chosen settings (300 µm depth of the prototype flow-through cell) limited the upper detection to an OD value of 4 (a cell dry weight of ca. 2 g/l). The growth detection achieved on-line by the so-called TA (total absorption) and BCA (background corrected absorption) algorithms is shown in Fig. 6. Note that the growth detection algorithms are based on absorption measurements (pixel intensity). Hence, reducing the liquid depth inside the flow-through cell decreases the overall absorption and thus increases the upper detection limit of biomass that can be measured.

For that reason, a new generation of flow-through cells was developed whose depth can be adjusted automatically, allowing the system to handle a wider range of cell concentrations during the fermentation process. Secondly, a dual pump flow controller for automatic dilution was developed (ParticeTech ApS). In this way, samples from the fermenter can be automatically diluted providing an appropriate cell-concentration or, respectively, image quality for segmentation. This, together with the height (depth) adjustment of the flow-through-cell, simplifies tremendously the acquisition of on-line image data over a much larger range of cell concentrations.

Recent technological advances such as the oCelloScope instrument deliver information about both, the cell concentration and the morphology dynamics regarding cell size, the cell size distribution and the distribution between single cells, budding cells and cell clusters. The latter may for example be correlated with the production of insulin, which is highly relevant for insulin production processes based on yeast. Besides, studying the effect of relevant process events frequently challenging process operation, such as failure of stirring or aeration, on the cell morphology might bring significant benefits for integrated troubleshooting. Such findings may lead to novel, image-based monitoring strategies at production scale.

### Case study 2: real-time particle monitoring

**Fig. 7:**
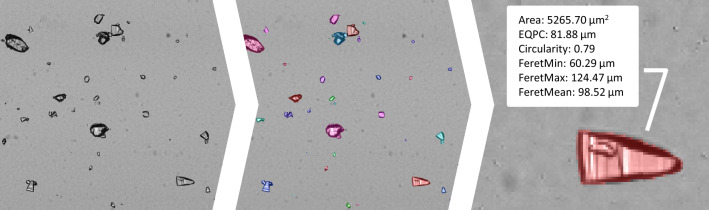
The process of image analysis, from imaging (left), through segmentation (middle) to analysis of individual particles (right). The particles are given random colors to indicate the particles detected during image segmentation

In the last decade, there has been significant developments within digital imaging, image analysis algorithms and computational processing power. This has allowed for the development of new real-time direct particle analysis methods, where high-resolution microscopic imaging can be used to capture images of particles in liquid suspension. By applying an image segmentation algorithm and subsequently analysing the identified particles, one can obtain information on particle population properties in a matter of seconds, including particle shape-, size- and morphology-distributions. The process of particle analysis using image analysis can be seen illustrated in Fig. 7.

A number of commercial particle monitoring solutions have become available in the last two decades. This includes in-line probe-based sensors (Mettler Toledo ParticleView [103], SOPAT [104]) but also non-invasive on-line flow-cell based sensors (Sympatec [105], ParticleTech [106]). The latter technology has the benefit of a more controllable imaging environment that improves image quality, which also heavily affects the measurement accuracy. This includes decreased background noise due to thinness of the flow-cell, improved lighting sources etc. Many of the commercial solutions have a lower detection limit down to a particle size of 0.5 μm. This allows for studying various chemical and biochemical processes that have particles in suspension. For instance, it is possible to study eukaryotic cells in fermentations, crystals in crystallizations, flocs in flocculation etc. With sampling times of less than a minute, one can now observe the process dynamics related to the particles and furthermore use the particle analysis as data source in process control strategies.

Nielsen et al. [107–110] have recently analyzed a number of different particle processes, including a lab-scale crystallization, an industrial scale crystallization and a lab-scale flocculation. Here they have used on-line and at-line sampling respectively, and analyzed the particles using the ParticleTech solution [106]. For the on-line measurements, liquid samples were withdrawn from the reactor using a peristaltic pump to an 800 µm thick flow-cell inside the Particle Tech microscope unit. After imaging, the liquid samples were returned to the reactor. For at-line measurements, samples were withdrawn manually from the process tank to a microtiter plate, which was then placed in the microscope unit for imaging. By sampling every 4–5 min, it was possible to capture the process dynamics as a set of time-series data of particle properties and particle concentration.

**Fig. 8:**
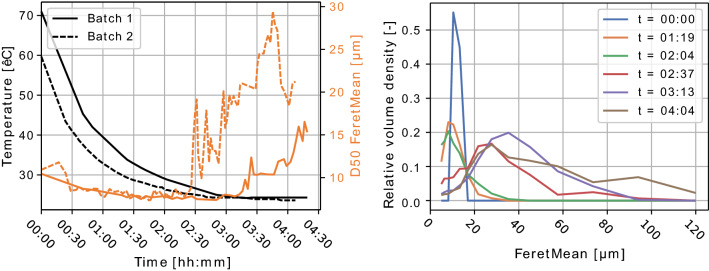
Particle size attributes from a lactose cooling crystallization, using data from Nielsen et al. [108]. Left: Reactor temperature and median (D50) Feret Mean particle size for two batch crystallizations. Right: Relative volume density of crystals in batch 2 for selected samples during the batch crystallization

**Fig. 9:**
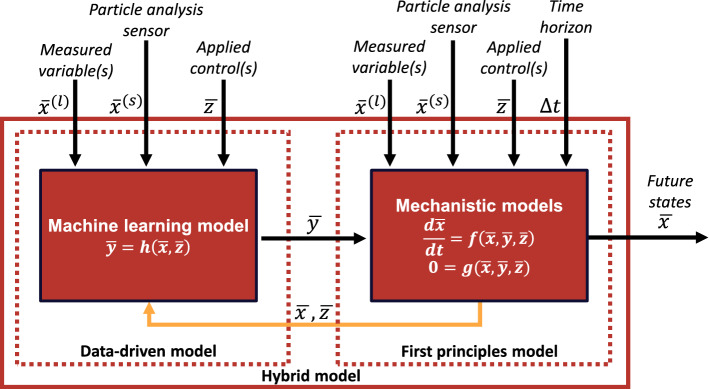
Hybrid model structure by Nielsen et al. [107] where they have used a deep neural network as the machine learning model. $\stackrel{-}{x}$ represents the state variables, $\stackrel{-}{z}$ represent the control actions and $\stackrel{-}{y}$ represent the kinetic rates. Reprinted from Computers and Chemical Engineering, Volume 140, RF Nielsen, N Nazemzadeh, LW Sillesen, MP Andersson, KV Gernaey, SS Mansouri, Hybrid machine learning assisted modelling framework for particle processes, 106,916, 2020, with permission from Elsevier

Data from a lab-scale batch cooling crystallization, presented in the work by Nielsen et al. [103], is illustrated in Fig. 9. Two batch crystallizations were carried out with slightly varying cooling profiles, resulting in varying particle size distributions. This can clearly be seen in the differences in median diameters (D50 FeretMean) at the end of the two batches, illustrated in the plot to the left. From the plot to the left in Fig. 8 it is also easy to see how the crystals are growing throughout the batch crystallizations, starting from an almost uniform distribution to a wide distribution.

As the mentioned particle analysis sensors can provide relatively frequent measurements, it becomes possible to capture intermediate process dynamics. This also makes it feasible to use more data-driven and complex kinetic models to improve the model prediction accuracy.

Nielsen et al. [107, 108] have recently proposed a hybrid modelling approach that accommodates the increased quantity of data available from real-time particle analysis sensors. Here they suggest a model structure combining first-principles mass and population balance models with a data-driven neural network model for estimating the process kinetics. Their model structure can be seen in Fig. 9. The inputs to the data-driven model consist here of multi-dimensional data from additional at-line/on-line/soft-sensors. The output of the data-driven model is a number of kinetic rates of particle phenomena, such as nucleation, growth, shrinkage, agglomeration and breakage rates. These rates are then included into a discretized first principles population balance model.

**Fig. 10:**
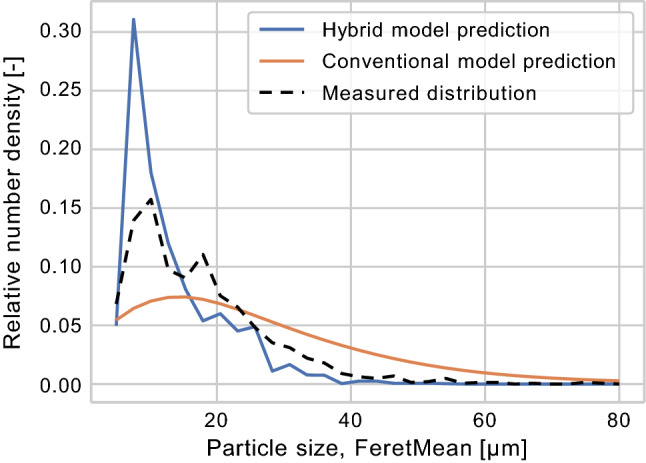
Conventional model compared to hybrid model [108]. Reprinted from Computers and Chemical Engineering, Volume 140, RF Nielsen, N Nazemzadeh, LW Sillesen, MP Andersson, KV Gernaey, SS Mansouri, Hybrid machine learning assisted modelling framework for particle processes, 106,916, 2020, with permission from Elsevier

The neural network here substitutes the conventional case-specific kinetic expressions. The conventional kinetic expressions typically contain one to five model parameters that are estimated using small amounts of experimental data, and typically only rely on a few process variables. For instance, for a crystallization such as the one presented in Fig. 10, one would typically only use the relative super-saturation, calculated based on the reactor temperature and solute concentration. Using a neural network instead, the number of input process variables can easily be extended beyond two process variables, but also reduced for systems with only limited process knowledge and/or lack of measured process variables.

The hybrid model structure was implemented by Nielsen et al. [108] using Tensorflow [111], an open source python software library. Automatic differentiation was here employed to significantly speed up the training of the hybrid model, which opens up for training the hybrid model during process operation, utilizing the latest process data.

Nielsen et al. [108] compared the hybrid model structure in Fig. 9, using the lactose case study data presented in Fig. 8, where temperature was used as the only measured process variable to model nucleation and growth rates, with a conventional, mechanistic model. They trained/fitted the hybrid model and a corresponding conventional crystallization model respectively using data from batch 2 and carried out end-to-batch simulations of the validation batch (batch 1) using both models. The resulting predictions can be seen in Fig. 10.

Nielsen et al. [108] concluded that the hybrid model was capable of capturing slightly more of the phenomena dynamics than the conventional model. There were however still discrepancies between the hybrid model and the final measured distributions, which was explained by the lack of training data. They also presented two additional case studies, on flocculation/breakage of silica particles and a pharmaceutical crystallization respectively, which both showed good results where only limited prior process knowledge was available.

One should note that the model complexity still needs to be decided based on the quality and quantity of experimental data, as an overly complex model will lead to over-fitting and a too simple model will lead to under-fitting. The increased data coming from the new real-time monitoring methods do however allow for using models with increased complexity without over-fitting, which in the end results in higher prediction accuracies.

### Case study 3: monitoring and closed loop control of cellulosic fermentations

Using non-refined natural substrates such as lignocellulosic material for the production of renewable fuels or chemicals has been central in the transition towards a sustainable economy. The main operational challenges associated with lignocellulosic material are the presence of mixed carbon sources (including C6 and C5 sugars such as glucose and xylose, respectively), the presence of potent inhibitors derived from the pretreatment of the biomass, and the high batch-to-batch variability of the substrates. Typically, cellulosic processes are run as fed-batch, where the concentration of substrates and inhibitors are kept below a certain level to avoid inhibitions and to increase the space–time yield. Control of the feed rate is a widely studied and often complex challenge in fermentation processes due to the non-linear nature of biological kinetics and to the limited ‘real-time’ data extracted from such processes. Using lignocellulosic feedstocks as a substrate for the fermentation increases this challenge due to its inherent natural variability. Not accounting for such variations often results in productivity losses and raises scheduling issues in the down-stream operations. Therefore, *real-time* monitoring methods are needed to implement advanced control schemes, whereas implementing better controls inevitably leads to improved operations with quantifiable benefits [112].

#### Open-loop data-driven monitoring of cellulose to ethanol fermentations

**Fig. 11:**
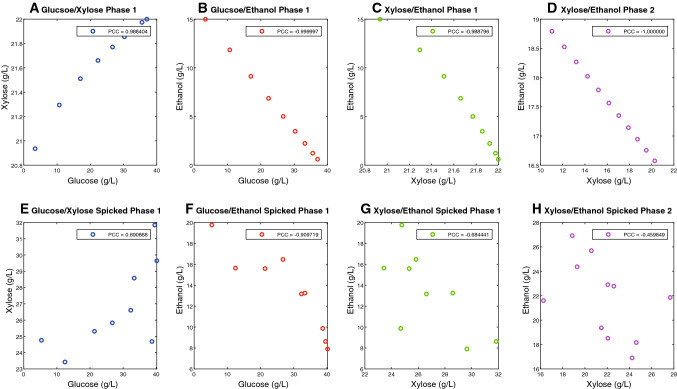
Pairwise correlation between the profiles of glucose, xylose, and ethanol in cellulose-to-ethanol batch fermentations in non-spiked (**a–d**) and spiked samples (**e–h**). **a–c** and **e–g** correspond to correlations occurring during the glucose consuming phase. **d** and **h** Correlations during the xylose consuming phase

When selecting a real-time monitoring scheme for fermentation processes, it is crucial to account for the ease of implementation and for how the information can be used to implement control strategies [113]. In cellulose-based fermentations, the commonly monitored variables such as pH or pO_2_ are easy to implement, but they do not deliver actionable information to develop control loops. Data-driven models are used to find correlations between the collected spectra and the concentration of the different analytes of interest. Linear models such as partial least squares (PLS) regressions are often used to model spectral data making use of the linear correlation between the analyte concentration and the absorbance in the spectra defined by Lambert Beer’s law. Training such algorithms to monitor fermentation processes efficiently is challenging due to the many sources of variation and the correlations between analyte concentrations occurring during the fermentation. In cellulose-to-ethanol fermentations, *S. cerevisiae* mainly consumes glucose and xylose to produce ethanol, CO_2_, and biomass. However, to sustain its growth, yeast also takes up nitrogen or vitamins and produces other byproducts such as glycerol or acetate. Moreover, during the initial stage of the fermentation, *S. cerevisiae* detoxifies the inhibitors present in lignocellulosic hydrolysates. All these changes in the composition of the media caused by compounds that are not analyzed can be summarized under the commonly used term matrix effects. Ideally, a data-driven algorithm, trained to monitor fermentation processes, should measure the concentration changes of the analytes of interest (e.g., glucose, xylose and ethanol for the case of cellulosic fermentations) independently from concentration changes in other compounds or from matrix effects. Per se, purely data-driven models (such as PLS regression) do not explicitly account for the dynamics of the fermentation, meaning that they will not be able to differentiate the effect of two analytes with correlated dynamics independently. This situation raises a challenge due to the high correlation between the dynamics of many compounds in fermentation processes. That is, in the case of cellulose-to-ethanol fermentations, the uptake of glucose or xylose is linearly correlated to the production of ethanol. This situation is exemplified in Fig. 11a–d for a typical cellulose-to-ethanol fermentation. Decoupling such correlations during the calibration (or training) of such models is crucial to reach reliable predictions even when the dynamics of the fermentation change. A common and efficient approach consists of taking samples at different times during the fermentation, and spiking them with different amounts of the analytes of interest to attain partially uncorrelated samples [114] (Fig. 11e–h).

Systematic approaches to plan the calibration sets based on design of experiments (DoE) can successfully be implemented in systems where the fermentation matrix can be isolated from the rest of the analytes [115]. This allows to completely decouple the concentrations of the different analytes and to distribute the samples evenly along the design space.

While PLS models allow estimating the concentrations of glucose, xylose and ethanol at each time point they have limited forecasting power and cannot be used to predict the evolution of the fermentation. However, this information is very valuable to determine the end-point of the fermentation and to schedule the downstream operations. Cabaneros et al. [115] used a hybrid framework to incorporate the PLS predictions of glucose into a mechanistic model of the fermentation to obtain high fidelity predictions of the progress of the fermentation.

#### Closing the loop: feedback control of cellulose-to-ethanol fermentations

**Fig. 12:**
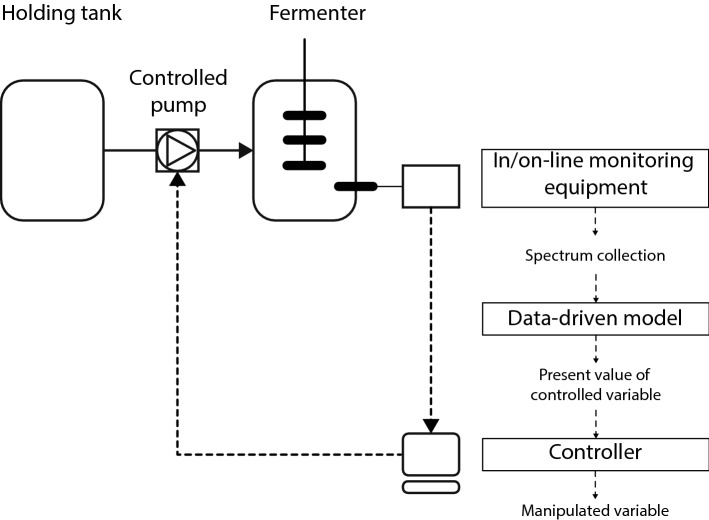
Schematic representation of a feedback control loop of the feed rate

The objective of feed rate control in cellulose-to-ethanol fermentations is to keep the concentration of inhibitors or glucose at a certain set point to avoid inhibitions or catabolite repression. In a feedback control scheme the measured variable (e.g., the concentration of inhibitors or substrate predicted using data-driven models) serves as input to a controller (such as a PID controller) to generate a signal back to the manipulated variable (e.g., the controlled pump) to adjust the feed rate (Fig. 12). To implement robust real-time monitoring schemes for fermentation processes, it is fundamental to thoroughly understand the behavior of the monitoring method and the dynamics of the fermentation or in any other unit operation as illustrated in [116, 117].

## Perspectives and barriers to implementation

The following perspectives and barriers to implementation are identified after analyzing in detail the case studies presented together with the current state-of-the-art in bioprocess monitoring and operations.

### Sensing technologies

The key to transitioning the current bio-based production processes into data-driven operations is the ability to gather information-rich production data in real-time. This is the primary objective of all three of the case studies presented and the focus of current efforts of the whole community. Such efforts aim at the development of sensing technologies and capabilities that go beyond the traditional temperature, pressure and flow rate measurements, as they provide information-rich process data that facilitate the data-driven operations of the future. However, these sensing technologies (including the ones that are mentioned in the case studies) require further development for them to be ready for long term use in industrial operations. Although the technologies have been proven on industrial trials and operations, their technology readiness level (TRL) and maturity require further improvement before being robust for long term operations in an industrial setting. In addition, peripheral supporting technologies are also under development that enable the use of these emerging technologies in an industrial bio-based production environment. These range from chemometrics and ML algorithms to GMP approved sampling ports and sample transfer systems.

### Human element and organizational readiness

In comparison to the chemical industries, operators in the biomanufacturing industry carry out a significant number of complex time critical tasks. If data-driven concepts are to improve these operations in the future, careful considerations must be given to the development of human machine interfaces (HMI’s) that effectively communicate the outcomes of these data-driven methods. In addition, such methods must pay heed to the human limitations when suggesting operational actions. For example, if a continuous addition to a process over an extended period is considered optimal, this turns out to be an infeasible task for an operator. Rather, this operational action might need to be modified to one addition to the process at an optimal time period.

From an organizational point of view, the operators and chemists are familiar with operating complex bio-based processes based on off-line sampling and fixed process recipes. These operational procedures, in many instances, ensure that the products meet quality specifications but pay no attention to product and efficiency losses. The introduction of data-driven operations will require that the operators and chemists adopt to dynamic schedules and on-line sampling methods, while embracing concepts, such as “real-time” product release, which are enabled by data-driven operations. This requires trust in data-driven operations by the operators, chemists and technical managers, and careful management of its implementation. The level of trust can be increased by improving the transparency of the data-driven operations and explicitly disclosing the underlying assumptions and algorithms used.

### Value proposition

With the current Industry 4.0 push that has been felt by the management level, there is increased demand for technologies that fit these criteria. But, to achieve truly smart (bio)manufacturing, noticeable fund allocations will be required from the corporate management that are more used to investing in “steel” for increased capacity (e.g. new equipment and/or new plants). These funds need to transition the current technologies in sensing, process monitoring and closed loop control to methods and technologies that are sufficiently robust for industrial operations. This also requires that the management organization at all levels is committed to make such investments. This further implies that the development and implementation cost of all these technologies must be outweighed by the benefits in terms of efficiency, throughput and quality, as well as the environmental footprint.

### Regulation

There are strict regulatory hurdles for the implementation of new technologies in the bio-pharmaceutical and food ingredients industries which compose a significant part of the biomanufacturing industry. The current regulations require operations to follow a strict recipe, whereas the value proposition for these technologies is that they are to adapt the recipes based on “real time” information so as to allow for improved operations (throughout, quality, efficiency, resource usage). This warrants a change in regulations following a QbD approach which has already been proposed, where a “region” of process operations is allowed as opposed to strict recipes.

## Conclusion

The promise of improved efficiency brought by smart biomanufacturing has sparked the interest of both academia and industry towards the development of smart technologies. However, the actual implementation of these technologies in an industrial setting will require significant further efforts. As illustrated by the first two case studies, there are still major technical challenges that must be overcome to ensure that sufficiently large data sets can be collected. These two case studies also highlight the ability to adopt already developed ML algorithms so as to accelerate the implementation of specific process monitoring needs. The last case study demonstrates how fully automated closed loop control can be achieved in a fermentation operation by leveraging large amounts of information-rich process data. As detailed in the perspectives section, there are significant barriers that must also be addressed alongside the development of the core sensing, monitoring and control technologies. All in all, this manuscript illustrates that there are exciting ongoing developments in the sensor area for monitoring and control of bio-based processes that hold the promise of moving the whole industry towards smart biomanufacturing in the future.

## Abbreviations

AI: Artificial intelligence
BCA: Background corrected absorption
NAD(P)H: Nicotinamide adenine dinucleotide phosphate
FDA: Food and drug administration
FTIR: Fourier-transform infrared spectroscopy
GC–MS: Gas chromatography–Mass spectrometry
GFP: Green fluorescent protein
GMP: Good manufacturing practice
HMI: Human machine interfaces
HPLC: High-performance liquid chromatography
MIR: Mid-infrared spectroscopy
ML: Machine learning
MVDA: Multivariate data analysis
NIR: Near infrared
OD: Optical density
PAT: Process analytical technologies
PCA: Principal components analysis
PCC: Pearson correlation coefficient
PLS: Partial least squares
QbD: Quality by design
TA: Total absorption
TRL: Technology readiness level
UV: Ultraviolet
YFP: Yellow fluorescent protein
YPD: Yeast extract peptone dextrose
